# Condom use to enhance regression of cervical intraepithelial neoplasia: study protocol for a randomized controlled trial

**DOI:** 10.1186/s13063-019-3564-4

**Published:** 2019-08-02

**Authors:** Malene Skorstengaard, Julie Suhr, Elsebeth Lynge

**Affiliations:** 1 Department of Public Health, University of Copenhagen, Øster Farimagsgade 5, DK-1014 København K, Denmark; 2 Nykøbing Falster Hospital, University of Copenhagen, Ejegodvej 63, DK-4800 Nykøbing Falster, Denmark

**Keywords:** Cervical lesions, CIN2, condom use, regression rate

## Abstract

**Background:**

Condom use can reduce the risk of infection by human papillomavirus (HPV). Furthermore, it has been suggested that condom use can increase the regression rate of cervical lesions. In Denmark, women with cervical intraepithelial neoplasia grade 2 (CIN2) and a future wish to conceive are not treated immediately but are followed up by a gynecologist about 6 months later. The aim of this project was to determine if advising women to have their male partners to use a condom during sexual intercourse in the follow-up period can increase the regression rate of CIN2.

**Methods/design:**

This is a randomized clinical trial of women with CIN2. The intervention group was advised to use condoms between the date of diagnosis and the date of their follow-up visit. The control group received standard care. Cervical samples were tested for HPV. The primary endpoint will be the intention-to-treat analysis with the relative rate of CIN2 regression between the intervention group and the control group. Regression is defined as <CIN2 at the follow-up visit. In addition, a per-protocol analysis of the regression rate in women adhering to condom use compared with the control group will be performed. The secondary endpoint will be the HPV-clearance rate in the condom group.

**Discussion:**

If condom use for 6 months can enhance the regression of cervical lesions, then more women can be spared conization. This is an efficient treatment of cervical lesions but is associated with an increased risk of preterm delivery.

**Trial registration:**

ClinicalTrials.gov, NCT02907333. Registered on 14 September 2016.

**Electronic supplementary material:**

The online version of this article (10.1186/s13063-019-3564-4) contains supplementary material, which is available to authorized users.

## Background

In Denmark, women aged 23–49 years are invited to a cervical screening every 3 years and women aged 50–64 years every 5 years [1]. Liquid-based cytology samples are collected by their general practitioner [2], and women with severe abnormalities are referred directly to a gynecologist. Every year, almost 400,000 cytology samples are taken, of which 40,000 are abnormal [3], resulting in 25,000 biopsies and 6,000 conizations [4]. Control of cervical cancer via screening, therefore, implies that many women attend repeated control visits or undergo conization. In Denmark, conization is performed using the loop electrosurgical excision procedure [5].

An examination by a gynecologist includes a colposcopy, a biopsy, and collecting a cytology sample. If her histology is normal or she has cervical intraepithelial neoplasia grade 1 (CIN1), the woman will, according to national guidelines [6], be followed up by her general practitioner, who will collect a new cytology sample after 6–12 months. If the woman is diagnosed with CIN2 and has a wish to conceive in the future, the recommendation is a follow-up visit to a gynecologist for a biopsy after 6 months. Women with CIN2 and with no wish to conceive or postmenopausal women are recommended to undergo conization. Women with CIN3 are always recommended to undergo conization [6].

Persistent infection with high-risk (HR) human papillomavirus (HPV) is a necessary but not sufficient condition for cervical cancer [7]. HPV is the most common sexually transmitted infection. Almost 75% of sexually active women become infected at some point in their life [8]. Most women will clear the infection, but for some women the infection persists, and for a very small number of women, the infection progresses to cervical cancer [9].

Male condom use (or, simply, condom use) has been shown to confer considerable protection against HPV infection, though the protection is less perfect than for other sexually transmitted diseases [10, 11]. Furthermore, condom use has been hypothesized to improve the regression rate of cervical lesions [12]. Firstly, condom use may reduce HPV infection and reinfection, and thereby allow the immune system to repair a cervical lesion. Secondly, semen has an immunosuppressive effect, which is considered an advantage in the reproductive process, but when cervical lesions have developed, the absence of semen could benefit the cellular immune response [13–15]. Lastly, the latex used for condoms is a foreign material that could enhance the cellular immune response and contribute to regression of a cervical lesion.

Two small studies found an increased regression rate of cervical lesions when condoms were used in the short term [16, 17], but this finding was not supported by a small questionnaire-based study [18]. The studies were too small to provide conclusive evidence, and condom use cannot, therefore, be included in the current guidelines for the management of CIN.

### Objective

The purpose of the present randomized controlled trial was to provide further evidence for the effect of condom use on the regression of cervical lesions. The hypothesis was that the regression rate would increase if condoms were used consistently for 6 months between the time of diagnosis and the follow-up visit to a gynecologist.

## Methods/design

### Patient and public involvement

Focus group interviews were performed prior to the project start with women in the relevant age groups. They advised on the design of the project, the intervention, and the duration of the intervention. Study participants were not involved in the recruitment or the conduct of the study.

### Study design

**Fig. 1:**
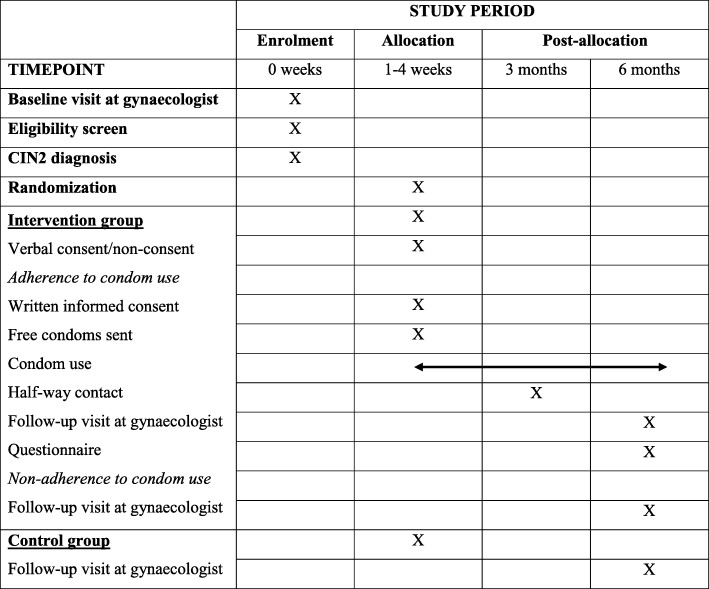
SPIRIT diagram of the project stages. CIN2 Cervical intraepithelial neoplasia grade 2

This was a randomized controlled clinical trial embedded in the routine diagnostic and follow-up visits to gynecologists for cervical lesions. It was a non-blinded study with a parallel allocation to an intervention group and a control group. The randomization ensured that there were two comparable groups of women, all following standard care according to national guidelines [6]. The intervention group were advised to use condoms to increase the regression rate of cervical lesions. The control group were not given this advice. Figure 1 is the SPIRIT diagram for the project.

### Target population

Inclusion criteria:

- Premenopausal women with histologically confirmed CIN2
- Aged 18 to 45 years
- A 6-month follow-up examination with a gynecologist has been scheduled

Exclusion criteria:

- Women with a latex allergy
- Women who are pregnant
- Women already in a follow-up program for a cervical lesion

#### Intervention group

Women in the intervention group are called by the project physician and informed about the project. They are advised always to use condoms during intercourse until the next follow-up visit with a gynecologist. If they have not been reached after two calls, a text message is sent. If they have not been reached after a further two calls and one text message, they are considered missing. Those contacted either consent or do not consent to participate verbally. Participant information and the informed consent form is sent to the home address of women who consent. Free condoms are sent to the woman once the signed informed consent form has been returned to the project physician. As a reminder about adherence, a text message is sent halfway through the intervention period. Participating women continue with their previous contraceptives if any. At the end of the intervention period, a questionnaire is sent by email. The questionnaire requests information on use of contraceptives, age at first intercourse, lifetime number of sexual partners, frequency of sexual intercourse, condom use in the intervention period, number of sexual partners in the intervention period, smoking, number and time of births and abortions, and HPV vaccination. The intention was to send the questionnaire to all women in the intervention group at the time of follow-up. However, the ethics committee of the Capital Region has stipulated that the questionnaire is sent only to women who have consented to condom use.

For women in the intervention group, their diagnosis at the follow-up visit with a gynecologist is retrieved from the pathology register [19]. For participating women, samples from both baseline and follow-up visits to a gynecologist are tested for HPV.

#### Control group

Women in the control group are not contacted by the project physician and therefore, not given advice on condom use as a way to enhance regression of their cervical lesions. Otherwise, they are examined and treated in exactly the same way as women in the intervention group. The biopsy result from the follow-up visit at the gynecologist is retrieved from the pathology register.

### Setting

The project takes place in three out of the five regions of Denmark. The project started in September and October 2016 in the Central Denmark Region and Region Zealand at both public hospitals and with gynecologists in private practice. The two regions were chosen because data from the pathology register showed that 700 study subjects would be expected per year, and the regions had relatively few stakeholders. To speed up recruitment, the project was expanded in the fall of 2017 to include some private practice gynecologists from the Capital Region.

In the Central Denmark Region, all hospitals and all gynecologists in private practice agreed to participate, but recruitment was not possible from two gynecologists in private practice due to logistical challenges. In Region Zealand, all hospitals and nine gynecologists in private practice agreed to participate. However, three gynecologists in private practice did not participate. It was not possible to recruit patients from one private practice due to their use of non-standard treatment. The Capital Region has four gynecology departments and 50 gynecologists in private practice. We asked 12 gynecologists in private practice to participate in the project, and five accepted (Table 1). The project physician has visited all gynecologists to provide standardized information.

**Table 1:** Number of gynecologists contributing to the project by region

Region in Denmark	Gynecologists in private practice		Hospital gynecology departments	
	Yes	No	Yes	No
Central Denmark Region	7	2	4	0
Capital Region	5	7	Not contacted	
Region Zealand	8	4	4	0

The gynecologists in hospitals and private practice send samples to the regional pathology departments for analysis. In Region Zealand and in the Capital Region, one pathology department per region is responsible for the analysis, while the responsibility is shared between departments in the Central Denmark Region. All pathology departments have agreed to send samples for HPV analysis.

All women included in the study are treated according to national guidelines. Women referred to a gynecologist after an abnormal cytology result undergo a colposcopy-guided biopsy. Samples are taken from sites with visible cellular changes and also from random sites. It is recommended that four or five samples are collected during each biopsy examination. The transformation zone in the cervical canal should be assessed with a cytobrush or by endocervical curettage. This procedure is recommended at the time of diagnosis and at the follow-up visit [6].

### Recruitment

**Fig. 2:**
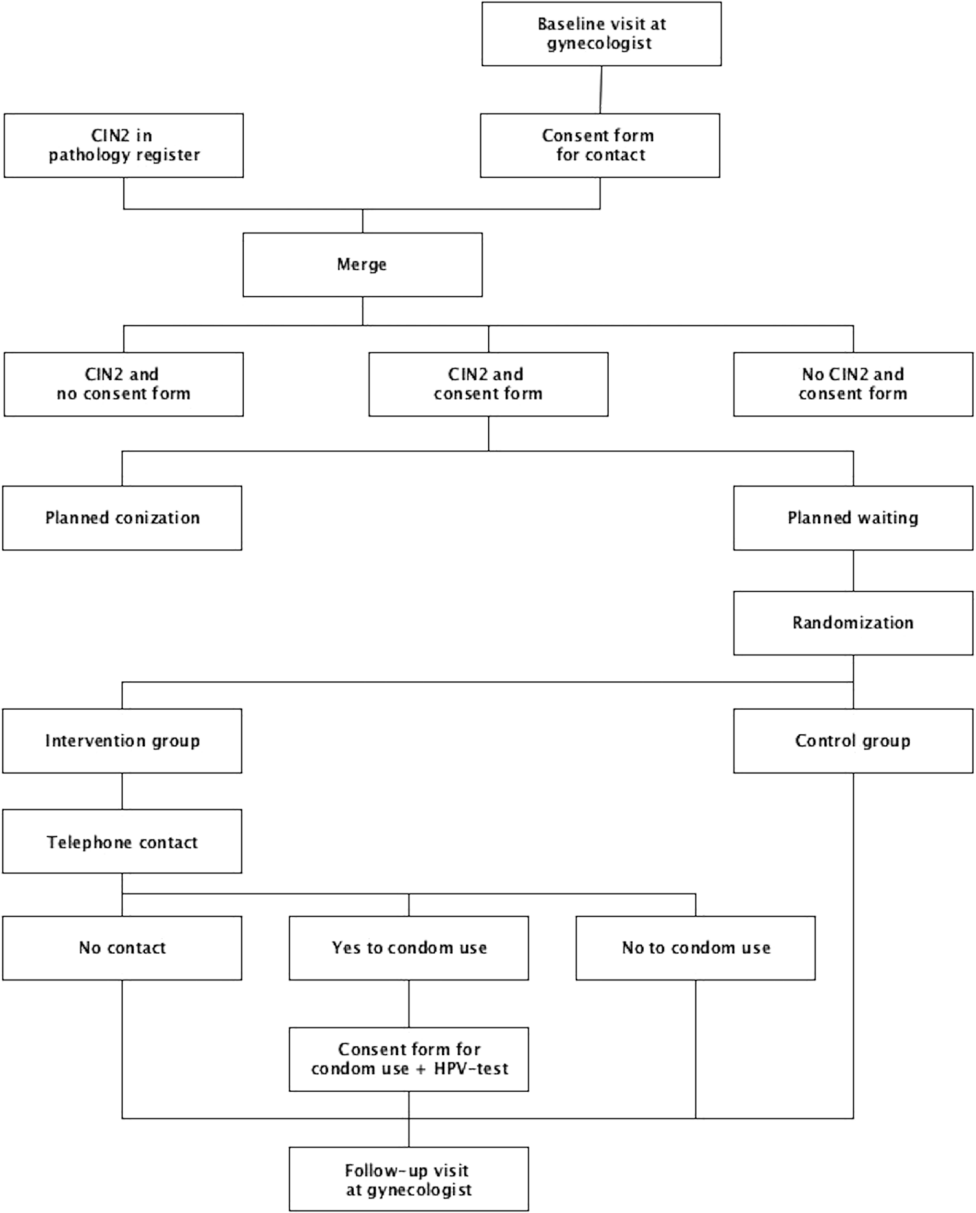
SPIRIT flowchart of the project design. CIN2 Cervical intraepithelial neoplasia grade 2

Women attending a participating gynecologist are asked for their phone numbers and to consent to be contacted by the project physician. Standard information about the project has been distributed by the project physician to the gynecologists to ensure standardized information is given to women. The consent forms are sent from the gynecologists to the project physician. The consent forms are matched with new CIN2 diagnoses retrieved from the pathology register every second week [19]. If there is a match between a consent form and a CIN2 diagnosis, the gynecologist is contacted for information about the treatment plan agreed with the woman. If watchful waiting with a follow-up biopsy in 6 months is planned, the woman will be included in the project and randomized (Fig. 2). No side effects, complications, or risks are expected due to participation in the project.

### Randomization

Women meeting the inclusion criteria and for whom we have received from the gynecologists a phone number and a signed consent form agreeing to be contacted are included in the study and randomized with an allocation of 1:1. The randomization sequence uses a computer-generated random number list per region, since policies for follow-ups and treatment vary slightly across the regions. Randomization is undertaken by a third person, after which the project physician contacts women randomized to the intervention group.

### Blinding

The trial is unblinded.

### Outcome measures

#### Primary endpoint

The primary endpoint is the relative rate of CIN2 regression between women in the intervention group and women in the control group. Regression is defined as having a diagnosis < CIN2 in the follow-up biopsy. As all women have CIN2 at entry, having less than CIN2 at follow-up entails a decreased risk of conization, and the woman will be referred back to her general practitioner for less frequent follow-ups according to national guidelines [6]. This outcome is, therefore, highly desirable. Another primary endpoint is the relative rate of CIN2 regression between women who consented to condom use and women in the control group. The regression rate in women who did not consent to condom use will be used to control for a possible selection bias. Regression rates will be reported by age.

#### Secondary endpoint

The secondary endpoint is clearance of HR-HPV for women who consented to condom use, based on the baseline sample and the follow-up sample. Clearance is defined as having a HR-HPV type at the time of CIN2 diagnosis that is not present at the follow-up. Previous studies have showed that having HPV16 will decrease the chance of regression of a CIN lesion [20]. Therefore, the initial HPV status and phenotypes might influence the regression rate of cervical lesions.

#### Other outcome measures

Predictors of adherence to condom use, CIN2 regression, and HR-HPV-clearance based on data on sexual habits etc., as known from the questionnaire.

### Power calculation

Based on the literature [16, 17], we expect the regression rate in non-condom users to be around 22%, and regression in condom users to be 2.5 times larger. We expect the rate of condom use to be 60% in the intervention group, which gives an expected relative risk of 2.0 for the intervention group compared to the control group. With a power of 95% and a 0.05 significance level, we would need 140 women in each group.

### Data analysis plan

The relative regression rate (RRR) for CIN2 in the intention-to-treat analysis will be calculated as

$$ \mathrm{RRR}=\frac{\left(\mathrm{number}<\mathrm{CIN}2\ \mathrm{at}\ \mathrm{follow}\hbox{-} \mathrm{up}\ \mathrm{in}\ \mathrm{in}\mathrm{tervention}\ \mathrm{group}\right)/\left(\mathrm{all}\ \mathrm{women}\ \mathrm{in}\ \mathrm{in}\mathrm{tervention}\ \mathrm{group}\right)}{\left(\mathrm{number}<\mathrm{CIN}2\ \mathrm{at}\ \mathrm{follow}\hbox{-} \mathrm{up}\ \mathrm{in}\ \mathrm{control}\ \mathrm{group}\right)/\left(\mathrm{all}\ \mathrm{women}\ \mathrm{in}\ \mathrm{control}\ \mathrm{group}\right)}. $$

The relative HPV-clearance rate (RCR) is calculated as

$$ {\displaystyle \begin{array}{l}\mathrm{RCR}=\\ {}\frac{\left(1-\mathrm{number}\ \mathrm{HR}\hbox{-} \mathrm{HPV}+\mathrm{at}\ \mathrm{follow}\hbox{-} \mathrm{up}\ \mathrm{in}\ \mathrm{in}\mathrm{tervention}\ \mathrm{group}\right)/\left(\mathrm{number}\ \mathrm{HR}\hbox{-} \mathrm{HPV}+\mathrm{at}\ \mathrm{baseline}\ \mathrm{in}\ \mathrm{in}\mathrm{tervention}\ \mathrm{group}\right)}{\left(1-\mathrm{number}\ \mathrm{HR}\hbox{-} \mathrm{HPV}+\mathrm{at}\ \mathrm{follow}\hbox{-} \mathrm{up}\ \mathrm{in}\ \mathrm{control}\ \mathrm{group}\right)/\left(\mathrm{number}\ \mathrm{HR}\hbox{-} \mathrm{HPV}+\mathrm{at}\ \mathrm{baseline}\ \mathrm{in}\ \mathrm{control}\ \mathrm{group}\right)}.\end{array}} $$

These calculations are possible because we have a closed population, in which all women recruited are followed up for the entire observation period. Logistic regression will be used to analyze the questionnaire data as predictors of outcomes. SAS version 9.4 will be used for the analysis.

### Data sources

From the gynecologists, we will receive the name, date of birth, and phone number of women who have consented to be contacted. Every second week, we will receive a list from the pathology register [19] with new CIN2 diagnoses. Sample results from the follow-up visits as well as HPV status at baseline and follow-up will be retrieved from the pathology register. Data on adherence to condom use will be obtained from the questionnaires. All data will be linked via the women’s unique personal identification number. The Danish Data Inspective Agency has approved the handling and storage of data (SUND-2016-21). All study-related material will be stored safely. Personal information on the participants is stored in secure computer systems.

### Dissemination

This study was registered at clinicaltrials.gov (NCT02907333) before recruitment started. This protocol article was written according to the Standard Protocol Items: Recommendations for Interventional Trials (SPIRIT) guideline [21] (Additional file 1, Reporting checklist for protocol of a clinical trial). The project design has been presented at meetings with the gynecologists. The project protocol has not been published elsewhere. All results will be analyzed and published in relevant international peer-reviewed journals. The results will be presented at international conferences, to the gynecologists, and to the health authorities. Furthermore, the results will be disseminated to the focus group that helped in planning the study, the study participants, and to the general public.

## Discussion

The use of self-care is increasing as an element of patient empowerment. Patients want to take part in decisions regarding their treatment and to contribute actively in optimizing the effect of treatment. For example, those with diabetes learn self-management through blood glucose monitoring [22, 23], those with chronic obstructive pulmonary disease learn how to use an inhaler [24], and those with inflammatory bowel disease learn how to monitor for an upcoming outbreak and when to start medication [25].

According to the present guidelines from the Danish Society of Gynecology and Obstetrics [6], the decision on whether to follow up or to treat CIN2 immediately is made together with the woman. It considers whether she wishes to conceive in the future. If the decision is made to follow-up only, no action is taken before the next visit with the gynecologist after 6 months and any regression relies entirely on the ability of the woman’s immune system. Our project tests a new way for women to intervene actively in the follow-up period. If the short-term use of condoms as tested here works as intended, women will have a choice for self-care and be able to do something active to enhance the regression rate of their cervical lesions.

Condom use was tested earlier in two small studies. Hogewoning et al. [16] ran a randomized controlled trial and found a statistically significantly increased regression rate for condom users of 53% (*n* = 57) compared with 35% for non-condom users (*n* = 51). Munk et al. [17] ran a prospective cohort study, and found an increased regression rate for condom users of 55% (*n* = 20) compared with 22% for non-condom users (*n* = 150). We, therefore, expect to find an approximately 2.5 times increased regression rate in the condom group compared with the control group [16, 17]. If this new larger study supports the observations from Hogewoning et al. and Munk et al., we will suggest temporary condom use to be included in guidelines to enhance regression of cervical lesions.

### Strengths and limitations

A strength of this study is its design as a public health randomized controlled trial embedded in the routine treatment and follow-up program for women with cervical lesions. This gives us two groups of women who are comparable except for the intervention. It is, furthermore, a strength to have three regions included. In Denmark, health care is paid for by the government and is free of charge [26], but there are minor regional variations in clinical procedures, and recruiting participants from three regions ensures that there is a representative sample of women.

The number of drop-outs is a limitation in this project. We do not receive consent to be contacted from all women attending a gynecologist, some women in the intervention group cannot be reached by phone or text message, and some women do not want to participate. Participating women may, therefore, be a selected group. Furthermore, adherence to condom use is inevitably self-reported. This information is collected from questionnaires at the end of the project, so there could be some recall bias. A punch biopsy may alter the HPV infection, and the disappearance of a type-specific HPV infection can be caused either by a biopsy or by an actual clearance. As the study is embedded in the clinical setting, we are not able to validate histology diagnoses or perform p16 staining. However, as the routine diagnostic procedures are used for both the intervention group and the control group, any possible misclassification will affect the two groups equally.

### Perspectives

If the project shows short-term condom use to enhance the regression rate of CIN2, this tool can be included in the guidelines for the management of patients with cervical lesions. Regression reduces the need for conization, which is associated with a risk of bleeding, infection, and severe adverse obstetric outcomes [5]. Shortening the time spent in control and follow-up will also improve the well-being of the women affected [27].

### Trial status

The recruitment of women with CIN2 started on 15 September 2016 and the final woman was recruited on 31 January 2019. The trial will end on 30 September 2019, when the last diagnosis from the follow-up will be made. Protocol version 4 dated 28 June 2016 has been approved by the ethics committee of the Capital Region.

## Additional file

Additional file 1: Reporting checklist for protocol of a clinical trial. (DOCX 30 kb)

## Data Availability

Not applicable.

## Abbreviations

CIN: Cervical intraepithelial neoplasia
CIN1: Cervical intraepithelial neoplasia grade 1
CIN2: Cervical intraepithelial neoplasia grade 2
CIN3: Cervical intraepithelial neoplasia grade 3
HPV: Human papillomavirus
HR: high-risk
RCR: Relative HPV-clearance rate
RRR: Relative regression rate
SPIRIT: Standard Protocol Items: Recommendations for Interventional Trials
